# ResNet Model Automatically Extracts and Identifies FT-NIR Features for Geographical Traceability of *Polygonatum kingianum*

**DOI:** 10.3390/foods11223568

**Published:** 2022-11-09

**Authors:** Yulin Xu, Weize Yang, Xuewei Wu, Yuanzhong Wang, Jinyu Zhang

**Affiliations:** 1 Medicinal Plants Research Institute, Yunnan Academy of Agricultural Sciences, Kunming 650200, China; 2 School of Agriculture, Yunnan University, Kunming 650504, China

**Keywords:** ResNet model, FT-NIR analysis, preprocessing, feature extraction, geographical traceability

## Abstract

Medicinal plants have incredibly high economic value, and a practical evaluation of their quality is the key to promoting industry development. The deep learning model based on residual convolutional neural network (ResNet) has the advantage of automatic extraction and the recognition of Fourier transform near-infrared spectroscopy (FT-NIR) features. Models are difficult to understand and interpret because of unknown working mechanisms and decision-making processes. Therefore, in this study, artificial feature extraction methods combine traditional partial least squares discriminant analysis (PLS-DA) and support vector machine (SVM) models to understand and compare deep learning models. The results show that the ResNet model has significant advantages over traditional models in feature extraction and recognition. Secondly, preprocessing has a great impact on the feature extraction and feature extraction, and is beneficial for improving model performance. Competitive adaptive reweighted sampling (CARS) and variable importance in projection (VIP) methods screen out more feature variables after preprocessing, but the number of potential variables (LVs) and successive projections algorithm (SPA) methods obtained is fewer. The SPA method only extracts two variables after preprocessing, causing vital information to be lost. The VIP feature of traditional modelling yields the best results among the four methods. After spectral preprocessing, the recognition rates of the PLS-DA and SVM models are up to 90.16% and 88.52%. For the ResNet model, preprocessing is beneficial for extracting and identifying spectral image features. The ResNet model based on synchronous two-dimensional correlation spectra has a recognition accuracy of 100%. This research is beneficial to the application development of the ResNet model in foods, spices, and medicinal plants.

## 1. Introduction

Medicinal plants currently have enormous research value [1]. In terms of medicinal use, most of them have multiple pharmacological activities, such as antibacterial, anti-inflammatory, and antioxidant effects [2]. The quality index of medicinal plants is the type and content of effective chemical components. The quality of medicinal plants directly affects their therapeutic effects. Geographical environmental factors are the key factors affecting the accumulation of medicinal phytochemicals [3]. Both Europe and China have geographical indication certification products, which recognise the quality in a specific geographical area. Therefore, it is of great significance to trace the geography of medicinal plants using fast and accurate analytical methods.

Chromatography, mass spectrometry, and metabolomics can accurately assess the quality of medicinal plants, but these methods are expensive, time-consuming, and highly specialised [4]. Near infrared spectroscopy (NIR) is a fast, reliable, and effective non-destructive analysis technique that can qualitatively and quantitatively evaluate the quality of medicinal plants [5]. However, NIR has highly overlapping peaks and broad absorption bands, and contains so much information that it is difficult to attribute and interpret them directly. The combination of NIR and chemometrics can overcome the above problems. Preprocessing is a crucial first step in NIR analysis, which removes random noise from the data and enhances spectral features of interest [6]. The NIR contains many non-informative variables, and so the feature extraction method often selects essential feature variables to describe the target properties [7]. The feature extraction of the spectra is usually located after preprocessing, and the preprocessing method often affects the feature extraction. Therefore, the combination of the preprocessing and feature extraction method is also the focus of the research.

Machine learning is a valuable data analysis tool, with robust computation and classification capabilities. Machine learning is already widely used for the authentication and traceability of food, spices, and medicinal plants [1,8,9]. The commonly used partial least squares discriminant analysis (PLS-DA) and support vector machine (SVM) models are excellent tools for analysing high-dimensional data [10]. Sun et al. used NIR and mid-infrared spectroscopy, combined with PLS-DA, SVM, the independent modelling of class analogy, and artificial neural network models to identify rhubarb accurately [11]. In a study on *Paris* species, both PLS-DA and SVM showed good model performance, and the PLS-DA model achieved 92% accuracy after mid-level data fusion [12]. With the development of big data, traditional learning models suitable for small data have certain limitations. Furthermore, traditional machine learning requires tedious preprocessing and feature extraction to achieve satisfactory results [13]. Deep learning models based on neural networks can be well adapted to the development of big data, and they have substantial advantages in image recognition and target detection [14,15,16]. Residual neural network (ResNet) is an excellent convolutional neural network (CNN) that can solve the vanishing gradient problem in deep learning models [17]. Deep learning can classify samples directly from spectrum images and automatically extract and identify the features of spectrum images [18]. However, the working mechanism and decision-making process of deep learning models are unknown, making it difficult to interpret and understand the models [19]. Yue et al. have previously investigated whether deep learning models are affected by the type and number of samples, and the results show that they are not [20]. Therefore, this study plans to understand deep learning models in preprocessing and feature extraction.

*Polygonatum* has been used to consume and treat diseases in China as early as 2000 years ago, and it has rich nutritional value and pharmacological effects [21]. Among them, *Polygonatum kingianum* is an officially designated edible and medicinal species. At present, the research on *P. kingianum* mainly focuses on its chemical constituents and pharmacological effects. There are few studies on the evaluation of its resource quality. Zhang et al. previously analysed the growth period of *P. kingianum* and concluded that after four years is the best harvesting period [22]. There are few studies on the geographical origin of *P. kingianum*, and the method of this study can provide a research basis for it.

To sum up, this study took *P. kingianum* as an example, and established the PLS-DA, SVM and ResNet models to trace its geographic origin. Artificial feature extraction methods combine traditional PLS-DA and SVM models to understand and compare the deep learning models. Furthermore, the impact of preprocessing on feature extraction was also explored. This study adopted four methods of feature variable extraction: the successive projections algorithm (SPA), competitive adaptive reweighted sampling (CARS), the variable importance in projection (VIP), and the number of potential variables (LVs). The most commonly used multiplicative scatter correction (MSC) and second derivative (SD) preprocessing for FT-NIR were employed. A linear PLS-DA and a nonlinear SVM model were chosen in order to understand the linear relationship of the extracted feature variables. In addition, the one-dimensional FT-NIR was converted into synchronous two-dimensional correlation spectra (2DCOS), in order to understand the ResNet model from the perspective of preprocessing. This research is beneficial to the application development of the ResNet model in foods, spices, and medicinal plants. 

## 2. Materials and Methods

### 2.1. Plant Materials and Preparation

**Figure 1:**
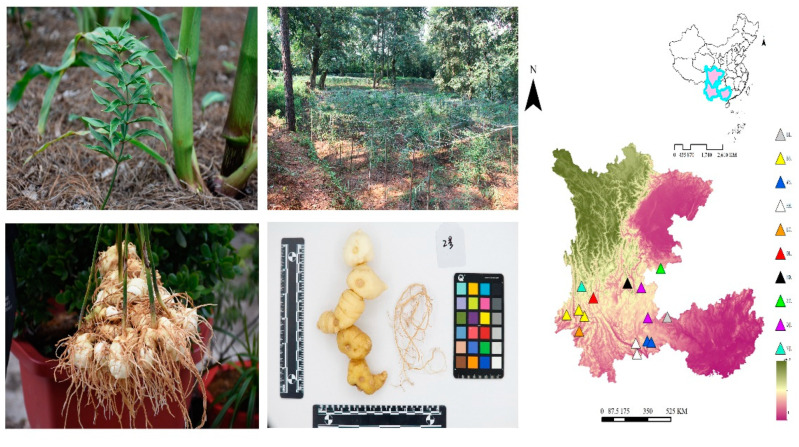
Basic information (plant, habitat and rhizome) and sampling location of *Polygonatum kingianum*.

A total of 207 *P. kingianum* samples were collected from 15 sampling sites in the Yunnan, Sichuan, and Guangxi provinces. It can be divided into 10 geographical sources based on geographic distribution. All samples were identified by Professor Jinyu Zhang (Institute of Medicinal Plants, Yunnan Academy of Agricultural Sciences, Kunming, China). The details of the samples are shown in Figure 1 and Table 1. After removing the aerial parts and fibrous roots, the samples were washed and sliced. They were then steamed in a steamer until utterly penetrated, and then dried in a 55 °C oven to constant weight. Subsequently, they were ground into powder, passed through a 100-mesh sieve, and stored in a labelled PE-sealed bag at room temperature, protected from light.

### 2.2. FT-NIR Acquisition

The FT-NIR spectrometer (Thermo Fisher Scientific Inc., Waltham, MA, USA) was used to scan *P. kingianum* powder. In the diffuse reflection mode of the integrating sphere, approximately 5 g of the sample was taken and placed in the sample cup each time. An accumulated 32 scans were performed in the range of 10,000–4000 cm^−1^, and the resolution was set to 8 cm^−1^ to obtain the spectral data. Each sample was measured 3 times, and the average spectrum was taken.

### 2.3. Data Processing

#### 2.3.1. Outlier Detection and Preprocessing

Anomalous samples are regarded as outliers, which may be caused by experimental changes or failures, and other uncertainties. The overall trend of the dataset may be completely changed by outliers [23]. So, outlier detection was first performed on the samples in this study. The data were visualised using a principal component analysis (PCA) model, followed by Hotelling’s T^2^ to detect outliers. The confidence interval was set to 95%, and samples of each class outside the confidence interval were removed.

Raw FT-NIR information often has unwanted variables or even erroneous information due to the measurement mode, sample state, and other external physical, chemical, and environmental factors [24]. Therefore, it is necessary to preprocess the data before analysis to reduce random noise and systematic variation in the spectral data, and enhance the spectral features of interest [6]. The most widely used preprocessing techniques for NIR are MSC and SD [25]. Therefore, the combination of MSC and SD is considered as a preprocessing method for FT-NIR. The preprocessed spectral data were compared with the original spectra in the later data analysis to confirm whether the preprocessing technique optimized the data.

#### 2.3.2. Feature Extraction

In fact, most spectral variables are usually related to some background phenomenon, and they are irrelevant in explaining the response variables. Feature extraction can improve prediction performance, make calibration reliable, and provide a more straightforward interpretation [26]. Therefore, four feature extraction methods were used for the feature extraction of FT-NIR. The model recognition ability without feature extraction, and different feature extraction methods were compared. (1) The VIP accumulates the importance of each variable reflected by weighting each PLS component, to screen important wavelength points [26]. Generally, variables with VIP scores of greater than 1 are characteristic variables [27]. (2) The LVs are selected through the PLS-DA model. The Q^2^ is a necessary index to evaluate the ability of the model to fit external data [28]. Therefore, the best number of potential variables is selected when Q^2^ reaches the maximum for the first time. (3) SPA is a variable selection technique that seeks to find spectral variables with the minimum level of collinearity. The selection principle of SPA is that the newly selected variable is the one with the largest projection value on the orthogonal subspace with the previously selected variable among the remaining variables [29]. (4) The CARS is a strategy to select the optimal combination of key wavelengths for multicomponent spectral data [30]. The most appropriate number of factors is selected when the root mean square error of cross-validation (RMSECV) value is the smallest.

#### 2.3.3. Two-Dimensional Correlation Spectroscopy (2DCOS) Conversion of FT-NIR

The deep learning model based on ResNet has unique advantages in image feature extraction and recognition [17]. Therefore, this study attempts to understand how these processing methods affect the deep learning model. In addition to the preprocessing of the original spectra, the FT-NIR were transformed into two-dimensional correlation spectra.

The two-dimensional correlation spectroscopy improves the spectral resolution by increasing the dimensionality, and it can effectively extract the characteristic information of weak peaks, migrated peaks, and overlapping peaks [31]. The generalised two-dimensional correlation spectra are calculated using the discrete generalised 2DCOS algorithm. According to Noda theory, the intensity of the dynamic spectrum is expressed as the vector *S* [32]:

(1) $$S\left(v\right)=\left[\begin{array}{c} s\left(v,t_{1}\right)\\ s\left(v,t_{2}\right)\\ \cdot \\ \cdot \\ \cdot \\ s\left(v,t_{m}\right) \end{array}\right]$$

In Formula (1), *v* is a variable, *t* is external perturbation (the difference between each sample spectrum and the average spectrum of each class), and *m* is the spectrum measured at *m* steps with equal intervals of perturbation *t*. The correlation intensities of *v*1 and *v*2 variables of synchronous two-dimensional correlation spectra are expressed as Φ (*v*1, *v*2):

(2) $$\Phi \left(v1,v2\right)=\frac{1}{m-1}S{\left(v1\right)}^{T}\cdot S\left(v2\right)$$

MATLAB 2017b software was used to automatically generate synchronous 2DCOS images. After removing abnormal samples, a total of 202 synchronous 2DCOS images (64 × 64 pixel) were generated. 

### 2.4. Data Analysis

#### 2.4.1. Partial Least Squares Discrimination Analysis

PLS-DA is a supervised linear classification model that extracts the difference information between samples and then performs classification. The PLS-DA model is suitable for a large number of variables, which can reduce the multiple linear effects of the variables [33]. Using the Kennard-Stone algorithm, the 202 samples were divided into 141 training sets and 61 test sets. The optimal number of LVs was selected based on the lowest RMSECV and high Q^2^ to build a seven-fold cross-validation model. The purpose of this was to select as many LVs as possible when the model does not fit well [34]. The model fit and robustness were evaluated according to the model fitting parameters R^2^ and Q^2^, root mean square error of estimation (RMSEE), RMSECV, and root mean square error of prediction (RMSEP). The classification performance and prediction performance of the model are reflected by the correct rate of the training set and test set. R^2^ and Q^2^ represent the cumulative explanatory power and cumulative predictive power of the model, respectively. The closer the value to 1, the stronger the robustness of the model. The closer RMSEE, RMSECV, and RMSEP are to 0, the stronger the model’s classification performance. To verify the fitting degree of the model, 200 iterations of permutation tests were performed to verify whether the model was overfitting according to its R^2^ and Q^2^ intercepts [20]. Typically, the R^2^ intercept is less than 0.4, and the Q^2^ intercept is less than 0, indicating that the model is not at risk of overfitting.

#### 2.4.2. Support Vector Machine

The nonlinear support vector machine model is a supervised model suitable for small sample classification. It uses the mapping method to map the sample data to a high-dimensional space to solve the optimal hyperplane to achieve classification [35]. The choice of the optimal penalty parameter (*c*) and kernel parameter (*g*) for the SVM has a significant impact on the classification accuracy [36]. The higher the c value, the lower the fault tolerance rate of the model, and the model is prone to overfitting; if the *c* value is too low, the model will be meaningless. Therefore, based on seven-fold cross-validation, this study used the grid search method to select the optimal *c* and *g*, and a nonlinear SVM model was established to trace the geographical origin of *P. kingianum*. Similar to the PLS-DA model, the KS algorithm was used to divide the training sets and test sets. Then, SVM models were built using different feature variables separately to explore the effectiveness of different feature extraction methods. 

#### 2.4.3. Residual Neural Network Model

CNN is an efficient method to extract deep image features with a strong generalization ability, and it is widely used in the detection and analysis of complex foods [37]. ResNet is an excellent CNN with strong correctness and convergence, which can solve the gradient disappearance and network degradation problems of traditional CNN models [38]. This study used the deep learning model based on ResNet built previously by the research group, and the specific introduction information can be found in the reference [17]. To explore the effect of preprocessing on the automatic extraction of important feature variables using the ResNet model, raw spectral images, preprocessed spectral images, and synchronous 2DCOS spectral images were used as model inputs. The 16-layer ResNet model is established with a weight attenuation coefficient λ of 0.001 and a learning rate of 0.01. Using the Kennard-Stone algorithm, the data with outliers removed is divided into a 60% training set (122 samples), a 30% test set (51 samples), and a 10% (19 samples) external validation set. The training set is input to the model, and the stochastic gradient descent method is used to find the optimal parameters and the lowest loss value. The number of epochs in the model represents the efficiency of modelling. The smaller the epoch, the more efficient the modelling. The loss value is used to explain the convergence effect of the model, and a value close to 0 indicates a good convergence effect. The test set is used to verify the final effect of the model, and the external validation set verifies the generalisation ability of the model.

### 2.5. Software

All preprocessing methods and PLS-DA model building were performed using SIMCA-P^+^ 14.0 software (Umetrics, Umea, Sweden). MATLAB R2017a software completed the generation of synchronous 2DCOS spectral images, and the establishment of the SVM model and ResNet model. The rest of the images were created using Origin 2021.

## 3. Results

### 3.1. Principal Component Analysis

**Figure 2:**
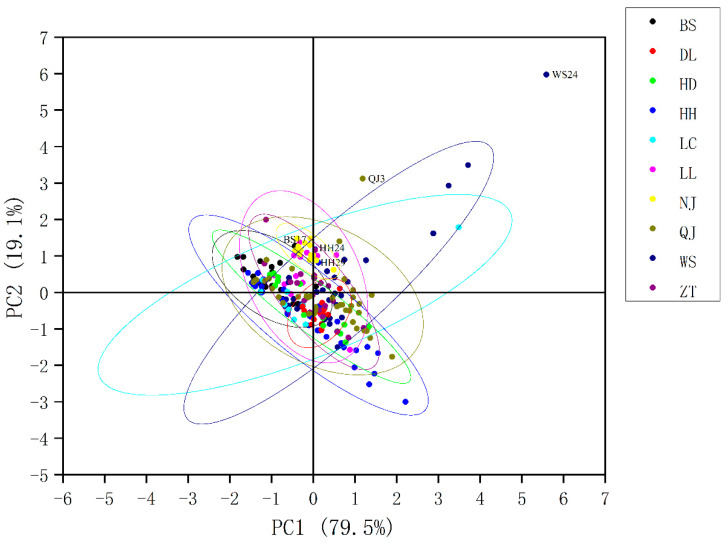
PCA analysis of FT-NIR.

This study used PCA to visualise the FT-NIR data, investigate the variability and distribution characteristics between samples, and remove outliers. The PCA score plot of the raw FT-NIR is shown in Figure 2. Ellipses represent 95% confidence intervals for each class, and samples outside the ellipse are considered abnormal. As shown in Figure 2, there were five abnormal samples. The sample numbers were BS17, HH24, HH29, QJ3, and WS24. In the subsequent analysis, removing outliers would be considered, to avoid affecting the accuracy of the analysis. PC1 accounted for 79.5% of the variance, and PC2 was responsible for 19.1% of the variance. The first two principal components explained 98.6% of FT-NIR information. However, the samples overlap, and the chemical information is highly similar. PCA cannot describe the differences between samples well, and it is not sufficient for the geographical traceability of *P. kingianum*. Establishing supervised machine learning models for more accurate traceability is necessary. 

### 3.2. FT-NIR and Pre-Processing Analysis

**Figure 3:**
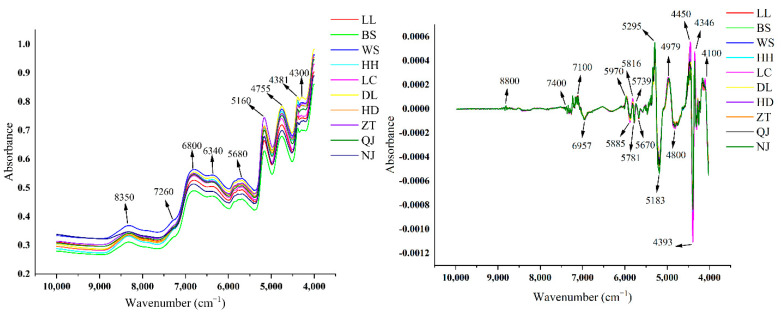
Average FT-NIR spectra, raw averaged FT-NIR spectra (**A**), MSC and SD preprocessing (**B**).

After removing outliers, the original FT-NIR characteristics of *P. kingianum* are shown in Figure 3. From the figure, the characteristic peaks are mainly concentrated at 7000–4000 cm^−1^ and 8500–8000 cm^−1^. FT-NIR can reflect the information on the organic composition containing hydrogen in the samples (N-H, C-H, O-H). The specific peak distribution is as follows: (1) The 4324–4260 cm^−1^ absorption bands are related to the C-H stretching and deformation combination; (2) approximately 4400 cm^−1^ is an O-H stretching and C-O stretching combination, possibly related to glucose; (3) approximately 4750 cm^−1^ is the combination of C-O stretching and O-H deformation; (4) around 5170 cm^−1^ is the combination of O-H stretching and the first overtone of C-O deformation, related to H_2_O and polysaccharides; (5) 6300–5400 cm^−1^ is the first overtone of C-H stretching; (6) the band around 6800 cm^−1^ is the first overtone of the O-H stretching vibration in H_2_O; and (7) the band near 8380–8230 cm^−1^ is the second overtone of stretching vibrations of C-H in the CH_3_ and CH_2_ groups [39,40,41]. The FT-NIR pre-processing with MSC and SD is shown in Figure 3B. The pre-processed FT-NIR showed more characteristic peaks, showing more differences at 4450 cm^−1^, 4393 cm^−1^, 4346 cm^−1^, and 4100 cm^−1^. Both the original and pre-processed spectra have similar characteristic peaks and differences in absorbance, but these differences are not enough to distinguish them intuitively. Therefore, machine learning was chosen for further analysis.

### 3.3. Variable Selection Analysis

**Figure 4:**
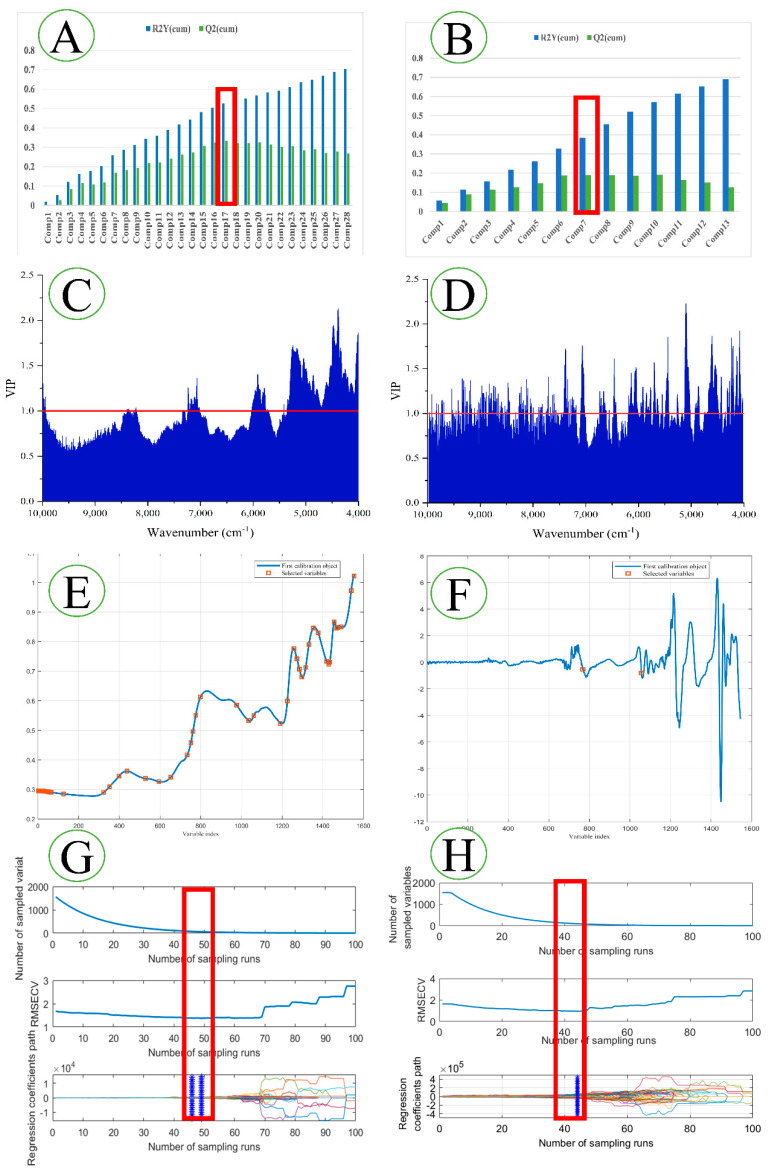
Four processes for manually extracting FT-NIR feature variables. (**A**,**C**,**E**,**G**) are the selection processes of the original spectra, (**B**,**D**,**F**,**H**) are the selection processes after preprocessing. (**A**,**B**) are LVs, (**C**,**D**) are for VIP, (**E**,**F**) for SPA, and (**G**,**H**) are for CARS. The red boxes represent the variables selected.

**Figure 5:**
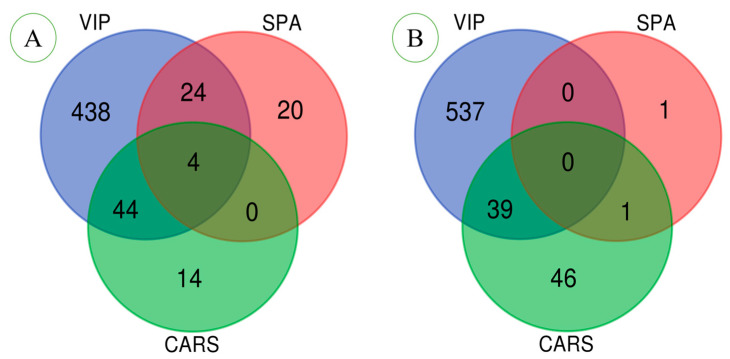
Venn diagram comparison of feature variables extracted using VIP, SPA, and CARS methods. Raw spectra (**A**), MSC + SD (**B**).

Variable selection can enhance the interpretability of FT-NIR data and remove irrelevant variables, thereby improving the efficiency and accuracy of machine learning. To explore the ability of machine learning to extract and identify FT-NIR features, four variable selection methods were used in this study. (Figure 4). The effects of preprocessing on feature extraction were also compared. The PLS-DA model was used to select the number of characteristic LVs when Q^2^ reached its maximum for the first time (Figure 4A,B). Without preprocessing, Q^2^ reached a maximum of 0.333 with 17 LVs; with MSC and SD treatment, Q^2^ reached a maximum of 0.190 with 7 LVs. At the same time, variables with a VIP value of greater than 1 were selected (Figure 4B,C). In total, 510 wavenumbers were selected as significant variables for the non-preprocessed FT-NIR, and 576 wavenumbers were selected for the preprocessed FT-NIR. Using the SPA algorithm, when the root mean square error (RMSE) of the model was the smallest, the optimal number of variables was selected (Figure 4E,F). The SPA algorithm was used to select 48 wavenumbers (RMSE = 1.2137) in the original FT-NIR, and 2 wavenumbers (RMSE = 2.0708) for the preprocessed FT-NIR. Using the CARS algorithm, the optimal number of variables was chosen when the RMSECV was the smallest (Figure 4G,H). A total of 62 wavenumbers were selected for the original FT-NIR, and 86 wavenumbers were selected after preprocessing. The different characteristics of the VIP, SPA, and CARS selection variables are shown in Figure 5. The MSC and SD preprocessing helped the VIP and CARS algorithms to extract more important variables, but SPA only proposed two variables, resulting in the loss of important variables. After preprocessing, fewer LVs were extracted. There are obvious differences in the methods of different variable selections. As for which method is better, further model verification is needed.

### 3.4. PLS-DA Classification Model

The PLS-DA model was established according to the selected characteristic variables. Table 2 lists the parameters and discriminant accuracy results for all models. It can be seen from the table that there are significant differences in the recognition abilities and model performances of the models established using different feature extraction methods. All models were subjected to 200 iterations of permutation tests, and no risk of overfitting was found (Figure S1).

From the perspective of extracting feature variables, the PLS-DA model established by screening VIP values of greater than 1 has the best classification performance and prediction performance, whether it is the original or preprocessed spectra. The model established by the original spectral screening VIP has an R^2^ of 1, the accuracy of the training set is 94.33%, and the accuracy of the test set is 86.89%. However, Q^2^ is only 0.28, indicating that the prediction excellence of the model is not good, which may be due to 510 important variables extracted using VIP. After preprocessing, the performance of the model established using VIP is significantly improved. Figure S2 shows the confusion matrix diagram of the VIP extraction model. From the confusion matrix, the model of class 5 is the worst, and the number of samples may be the reason. After preprocessing, the performance of the fifth category model is significantly improved. According to the correct rate of the test set, VIP (86.89%) > CARS (81.97) > SPA (78.69) > LVs (77.05) = Original (77.05). In general, the effect of the PLS-DA model established after the feature extraction is better than that without the feature extraction.

Regarding preprocessing, the FT-NIR preprocessed using MSC and SD is more accurate in the training set than the model established by the original spectra, but the accuracy in the test set is lower. Both R^2^ and Q^2^ are also lower than the original model. The reason may be that more spectral information is displayed after preprocessing, but the PLS-DA model cannot fit this information well. Whether the original or preprocessed spectra, the model effect established by the important variables extracted by VIP is the best. However, after preprocessing, the PLS-DA model established by the variables extracted using the LVs, SPA, and CARS methods has significantly lower recognition accuracy than the spectra without feature extraction. Especially with the SPA algorithm, only two important variables are proposed, which makes it challenging to retain important spectral information. After preprocessing, although more spectral information will be displayed, inappropriate feature extraction methods may lose important spectral information. This is not conducive to model construction and optimisation, resulting in poor model performance and a low recognition rate. In this study, the models established using the SPA, CARS, and LVs methods to extract the original and preprocessed spectral features could not accurately realise the geographical traceability of *P. kingianum*. Future research could focus on the correlation between different processing methods and feature extraction to better understand the spectral features.

### 3.5. SVM Classification Model

**Figure 6:**
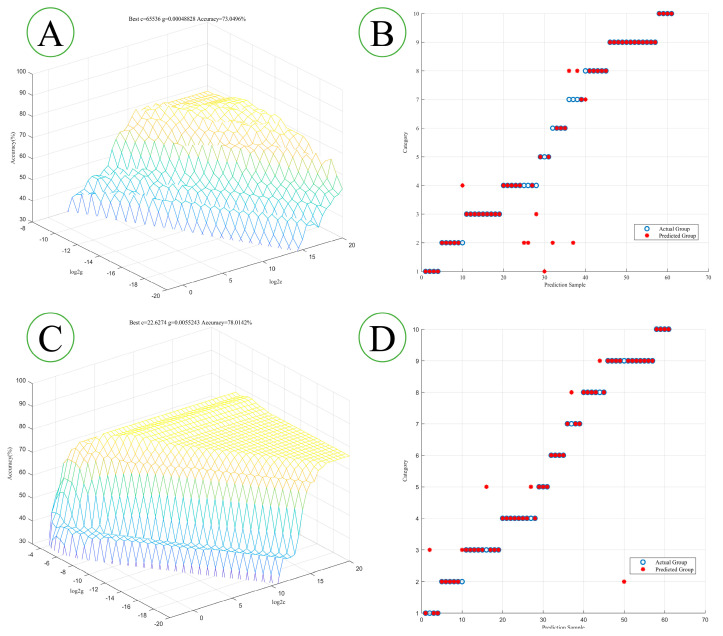
The optimal separation hyperplane of the SVM model based on VIP, and the classification results of the training set. Raw spectrum (**A**,**B**), MSC + 2D (**C**,**D**).

Table 3 shows the optimal *c* and *g* of all SVM models, as well as the accuracy of the training and test set. From the perspective of the feature extraction methods, the performances of models established via feature extraction are mostly better than those without feature extraction. The SVM model established using the original spectra without the feature extraction has a correct rate of 68.09% for the training set and 19.67% for the test set. Due to the large spatial dimension of the input, its *c* value is large, and there is a risk of overfitting, resulting in the low performance of the model. Therefore, suitable feature extraction methods are needed to select important variables from the spectral data to reduce noise, and irrelevant and redundant information, and thus reduce the dimensionality of the input space. As can be seen from Table 3, the models built with the variables extracted using VIP, SPA, and CARS show similar performances, with an accuracy of about 70% for the training set and of about 80% for the test set. The accuracy of the test set is higher than that of the training set, and the *c* value is enormous, so that the model may have the risk of overfitting. However, the SVM model established by LVs is an exception. Its training set accuracy rate is the highest among these models, but the test set accuracy rate is only 44.26%. The reason for this may be that there are too few feature variables to describe the different information between samples. Like the PLS-DA model, the model established by the VIP dataset has the highest classification accuracy, but is slightly lower than the PLS-DA model. Figure 6A,B shows the optimal separation hyperplane and classification results of the SVM model established on the VIP dataset without preprocessing. As can be seen from Figure 6B, the class 4 and class 7 test sets have the highest classification error rates.

After preprocessing, the performance of the model built on the dataset without feature extraction is significantly improved, with the *c* value becoming smaller and the *g* value increasing. It is worth noting that although more spectral information is displayed after preprocessing, the complexity of the model is significantly reduced, which is more conducive to the classification of samples. In terms of preprocessing, the performance of the models established by the VIP and LVs datasets was improved. However, the performance of the model based on the preprocessed CARS dataset has deteriorated. The SPA dataset with only two variables cannot build an SVM model. The above results show that the performance of the PLS-DA model is generally better than that of the SVM model. However, in general, the above two models cannot accurately realise the geographical origin of *P. kingianum*. 

### 3.6. Residual Neural Network Model

**Figure 7:**
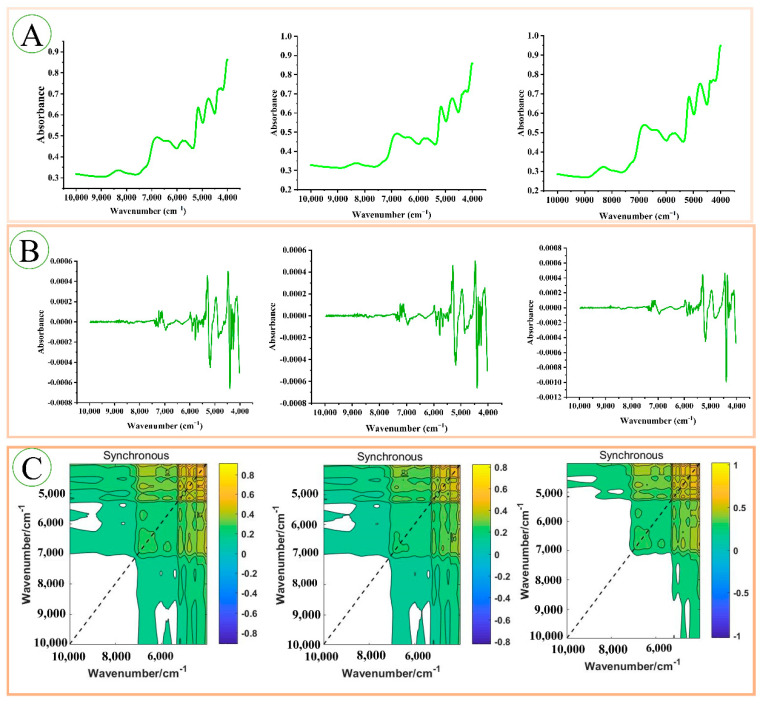
There are 3 data inputs for the ResNet model. (**A**) are the original FT-NIR images, (**B**) are the MSC and 2D preprocessed spectral images, and (**C**) are the Synchronous 2DCOS images.

**Figure 8:**
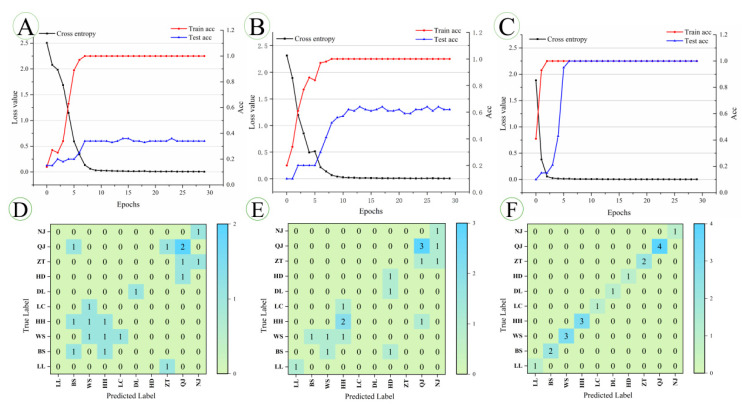
(**A**) (Original), (**B**) (MSC + SD), and (**C**) (Synchronous 2DCOS) are the accuracy curves and cross-entropy cost function curves of the training set and test set of the ResNet model. (**D**) (Original), (**E**) (MSC + SD), and (**F**) (Synchronous 2DCOS) is an externally validated confusion matrix.

Figure 7 shows the three kinds of partial image inputs of the ResNet model. A is the original spectra, B is the preprocessed spectral image, and C is the synchronous 2DCOS images. Figure 8A–C shows the accuracy curves of the training and test sets of the three ResNet models, as well as the cross-entropy cost function curve. The accuracy curve is used to evaluate the recognition ability of the model, and the cross-entropy cost function is used to reflect the convergence effect of the model. The confusion matrix for the external validation set is shown in Figure 8D–F. Table 4 summarises the detailed parameters of all deep learning models (epoch, loss value, correct rate on training set, test set, and external validation set).

According to the three ResNet models, the model based on synchronous 2DCOS images has the best recognition ability. Visually, the synchronous 2DCOS images display the most spectral information, showing colour information associated with peaks, as well as autocorrelated and cross-peaks associated with chemical composition. Therefore, it can be speculated that the ResNet model based on synchronous 2DCOS images extracts the most feature variables and has the most robust identification ability. The accuracy of the three ResNet models established in the training set can reach 100% with the increasing epochs, but the accuracy of the test set is different from each other. For the ResNet model established using the original FT-NIR image, when the epoch is 19, the correct rate of the test set is 34%, the correct rate of the external validation set is 36.8%, and the loss value is 0.009. After MSC and SD preprocessing, the accuracy of the test set is improved to 64% at 17 epochs, the accuracy of the external validation set is 47.4%, and the loss value is 0.009. The accuracy of the ResNet model established based on the synchronous 2DCOS spectral images of the training set and test set is 100% at the 10 epoch, and the loss value is 0.007. The accuracy rate of the external validation set is 100%, and 19 samples from 10 regions can be accurately classified. This model has the strongest generalisation ability. As the epoch increases, the loss values of the three models are all close to 0, which has a good convergence effect, indicating that the loss values of the models are not affected by preprocessing. However, the accuracies of the model test set and external validation set established by the original spectral and preprocessed spectral images are very low, which is insufficient to trace the geographic origin of *P. kingianum*. Comparing the three models, it can be found that the preprocessing is beneficial to the deep learning model for extracting and identifying the features of the spectrogram automatically. In summary, the recognition accuracy of the deep learning model based on synchronous 2DCOS is the best, and it is suitable for the geographical traceability of *P. kingianum*.

## 4. Discussion

**Figure 9:**
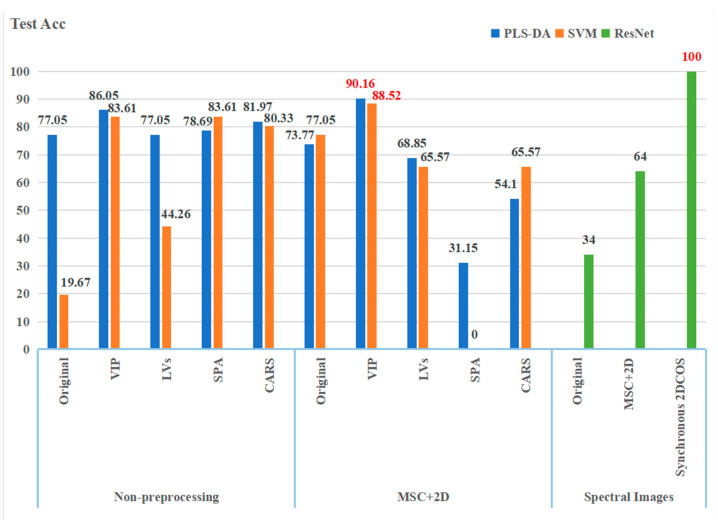
Comparison of PLS-DA, SVM, and ResNet models. The best results of each model are highlighted in red.

The PLS-DA, SVM, and ResNet models established based on different feature extraction methods and preprocessing methods have significant differences in recognition performance. Figure 9 shows a comparison of the three kinds of models.

From the perspective of the comprehensive recognition ability of the model, the recognition rate of the ResNet model based on synchronous 2DCOS spectral images is 100%. The highest recognition rates of the traditional PLS-DA and SVM models are 90.16% and 88.52%, which are not as good as the ResNet model. Comparing the PLS-DA and SVM models as a whole, the PLS-DA model has better feature variable recognition ability. This result means that the ResNet-based deep learning model has advantages over traditional machine learning methods in FT-NIR feature extraction and recognition. The deep learning model based on ResNet does not need to manually extract spectral features. Its working principle automatically extracts features and identifies them during spectral training.

From the aspect of feature extraction, the important feature variables and quantities extracted using different feature extraction methods are different (Figure 5). The VIP method extracted 576 variables from the original 1557 variables, the SPA algorithm selected 48 variables, and the CARS selected 62 variables. The three variable selection methods only select four common variables, and the feature variables extracted using the SPA and CARS algorithm are primarily different. The PLS-DA model selected 17 LVs. After MSC and SD processing, VIP and CARS extracted more feature variables, but the SPA algorithm only extracted one variable, resulting in the loss of important feature information. The PLS-DA model selects seven LVs. The PLS-DA model is a linear model, while the SVM is a nonlinear model. The performance of the SVM model established by the unprocessed FT-NIR data is inferior. It may be that the FT-NIR is highly overlapping and has strong collinearity, so that the SVM model cannot effectively extract and identify nonlinear characteristic variables. The high likelihood of LVs extracted from the raw spectra is also a linear variable. The PLS-DA and SVM models show little difference in the recognition accuracy of the feature variables extracted using the VIP, SPA, and CARS methods. However, after MSC and SD preprocessing, the recognition accuracy of the SVM model for uncharacterised spectra is significantly improved. It may be that after preprocessing, overlapping peak information is displayed, and the SVM model can extract nonlinear feature variables from it. After preprocessing, the VIP method extracts more feature information, and the model recognition rate is slightly improved. Although the CARS method also selects more feature variables, the performances of the two types of traditional learning models decreases significantly. The reason for this may be that the CARS method after preprocessing is not conducive to extracting differential information, and that there may be important variables that the SPA algorithm cannot effectively extract after preprocessing. The working mechanism of the ResNet model is unknown, and the extracted feature information is also unknown. Therefore, this study indirectly understands the feature variables extracted by ResNet from the preprocessing aspect. The ResNet model recognises only 34% of the raw FT-NIR. The spectra processed using MSC and SD show more spectral information, and the model recognition rate is improved to 64%. The recognition rate of the ResNet model established via synchronous two-dimensional correlation spectroscopy can reach 100%.

It can be seen from the results of this study that preprocessing affects the extraction of important features from the spectra. From the perspective of manual extraction methods, MSC and SD preprocessing are beneficial for VIP feature extraction, but not for CARS, SPA, or LVs extraction. The ResNet model is beneficial for extracting more important features from synchronous 2DCOS images, which contain more spectral information. Nevertheless, it is worth noting that the deep learning model based on the ResNet model extracts spectral image features, while the traditional learning model extracts spectral data features. Currently, the spectral features extracted by the ResNet model are unclear. Therefore, it is necessary to understand the important feature information extracted by the ResNet model from artificial feature extraction methods and preprocessing. This is conducive to interpreting the model and understanding the correlation between target parameters and feature information. 

## 5. Conclusions

In this study, the PLS-DA, SVM, and ResNet models were established to trace the geographic origin of *P. kingianum*. Using four extraction methods of VIP, CARS, SPA, and LVs, combined with the linear PLS-DA and nonlinear SVM model, the ability of the traditional model to extract and identify FT-NIR characteristic variables is discussed. The deep learning model based on ResNet can automatically extract and identify spectral features. This study aimed to understand the ResNet model from the perspective of preprocessing. Raw spectral images, MSC, and SD preprocessed spectral images, and synchronous 2DCOS images were used as data inputs to the ResNet model. The effects of preprocessing on the extraction and recognition of feature variables in traditional pattern recognition and ResNet models are discussed. The results show that preprocessing has a significant impact on feature extraction. After MSC and SD sum processing, the VIP and CARS methods are beneficial to the proposed feature variables, but the traditional model’s ability to identify the feature variables extracted by CARS has declined. After preprocessing, the SPA algorithm only proposes two feature variables, resulting in the loss of important information. In general, after preprocessing, the performance of the traditional model based on VIP feature variables is the best, but the performance of the models established using other feature extraction methods is lower than that of the models without feature extraction. After spectral preprocessing, the recognition rates of the PLS-DA and SVM models are up to 90.16% and 88.52%. For the ResNet model, preprocessing is beneficial for extracting and identifying spectral image features. After preprocessing, more informative features are displayed, especially for the model based on synchronous two-dimensional correlation spectroscopy, with a recognition accuracy of 100%. To sum up, the ResNet model has significant advantages over traditional models in feature extraction and recognition. The method used in this study can provide a robust analysis for the resource evaluation of medicinal plants, and can be extended to other studies.

## Figures and Tables

**Table 1:** The specific sampling information of *Polygonatum kingianum*.

Region	Collection Sites	Numbers	N	E
LL	Longlin, Baise, Guangxi Province	LL1-LL14	24°47′02″	105°20′24″
BS	Changning, Baoshan, Yunnan Province	BS1-BS14	24°50′01″	99°36′43″
	Yingjiang, Baoshan, Yunnan Province	BS15-BS17	24°54′52″	98°23′13″
	Longyang Banqiao, Baoshan, Yunnan Province	BS18-BS20	25°10′44″	99°13′37″
WS	Wenshan, Yunnan Province	WS1-WS12	23°29′01″	103°57′06″
	Bozhu, Wenshan, Yunnan Province	WS13-WS32	23°24′29″	104°13′22″
HH	Gejiu, Honghe, Yunnan Province	HH1-HH5	23°21′57″	103°9′50″
	Jinping, Honghe, Yunnan Province	HH6-HH32	22°46′59″	103°14′33″
LC	Yongde, Lincang, Yunnan Province	LC1-LC9	24°01′07″	99°15′33″
DL	Wase, Dali, Yunnan Province	DL1-DL12	25°50′07″	100°13′59″
HD	Huidong, Liangshan, Sichuan Province	HD1-HD14	26°38′26″	102°34′59″
ZT	Zhenxiong, Zhaotong, Yunnan Province	ZT1-ZT21	27°26′55″	104°52′33″
QJ	Xinjie, Huize, Qujing, Yunnan Province	QJ1-QJ20	26°23′49″	103°32′20″
	Secong Da, Shizong, Qujing, Yunnan Province	QJ21-QJ40	24°44′48″	103°59′31″
NJ	Lanping, Nujiang, Yunnan Province	NJ1-NJ13	26°27′30″	99°25′25″

**Table 2:** Parameters of PLS-DA Model Based on FT-NIR.

		R^2^	Q^2^	RMSEP	RMSEE	RMSECV	Train Acc (%)	Test Acc (%)
Non-preprocessing	Original	1	0.333	0.2246	0.2070	0.2315	87.23	77.05
	VIP	1	0.28	0.2080	0.1953	0.2436	94.33	86.89
	LVs	0.529	0.276	0.2246	0.2002	0.2547	87.23	77.05
	SPA	1	0.318	0.2216	0.2083	0.2332	90.07	78.69
	CARS	1	0.309	0.2199	0.2038	0.2339	88.65	81.97
MSC + SD	MSC + SD	0.618	0.191	0.2311	0.1923	0.2584	94.33	73.77
	VIP	0.712	0.302	0.2084	0.1780	0.2415	95.04	90.16
	LVs	1	0.292	0.2397	0.2259	0.2430	77.30	68.85
	SPA	1	0.104	0.2777	0.2739	0.2736	36.88	31.15
	CARS	0.666	0.182	0.2551	0.2336	0.2575	72.34	54.10

Note: R^2^ = Coefficient of determination; Q^2^ represents the prediction ability of PLS-DA model; RMSEP = Root mean square error of prediction; RMSEE = Root mean square error of estimation; RMSECV = Root mean square error of cross-validation; Train Acc = Classification accuracy of train sets; Test Acc = Classification accuracy of test sets.

**Table 3:** Parameters of SVM Model Based on FT-NIR.

		Best *c*	Best *g*	Train Acc (%)	Test Acc (%)
Non-preprocessing	Original	1048576	0.000061035	68.09	19.67
	VIP	65536	0.00048828	73.05	83.61
	LVs	2.8284	1	86.52	44.26
	SPA	1048576	0.0019531	70.21	83.61
	CARS	92681.9	0.00097656	72.34	80.33
MSC + SD	MSC + SD	64	0.00097656	69.50	77.05
	VIP	22.6274	0.0055243	78.01	88.52
	LVs	8	0.70711	79.43	65.57
	SPA	-	-	-	-
	CARS	22.6274	0.0625	61.70	65.57

Note: *c* = penalty parameter; *g* = kernel parameter; Train Acc = Classification accuracy of train sets; Test Acc = Classification accuracy of test sets.

**Table 4:** Parameters of ResNet Model Based on FT-NIR.

Dataset	Epoch	Loss Value	Train Acc (%)	Test Acc (%)	External Validation Acc (%)
Original	19	0.009	100	34	36.8
MSC + SD	17	0.009	100	64	47.4
Synchronous 2DCOS	10	0.007	100	100	100

Epoch is used to evaluate the modelling efficiency of ResNet. Loss value is used to assess the convergence of the ResNet model. Train Acc = Classification accuracy of train sets; Test Acc = Classification accuracy of test sets; External Validation Acc = Classification accuracy of external validation sets.

## Data Availability

Not applicable.

## Supplementary Materials

The following supporting information can be downloaded at: https://www.mdpi.com/article/10.3390/foods11223568/s1. Figure S1. Two hundred Permutation Iteration Tests for PLS-DA Models, without preprocessing (A,C,E,G,I), MSC + SD preprocessing (B,D,F,H,J), without feature extraction (A,B), LVs (C,D), VIP (E,F), SPA (G,H), CARS (I,J); Figure S2. Confusion matrix graph with the best performance in PLS-DA model (based on VIP feature extraction). A (training set without preprocessing), B (testing set without preprocessing), C (training set with MSC and 2D processing), and D (testing set with MSC and 2D processing).
