# Supplementary material for: ResNet Model Automatically Extracts and Identifies FT-NIR Features for Geographical Traceability of Polygonatum kingianum
Source: Foods. 2022 Nov 9;11(22):3568. doi: 10.3390/foods11223568 (PMC9689878; doi:10.3390/foods11223568)
Supplement: Supplementary file 1 [file foods-11-03568-s001.zip › foods-1947787-supplementary.pdf]

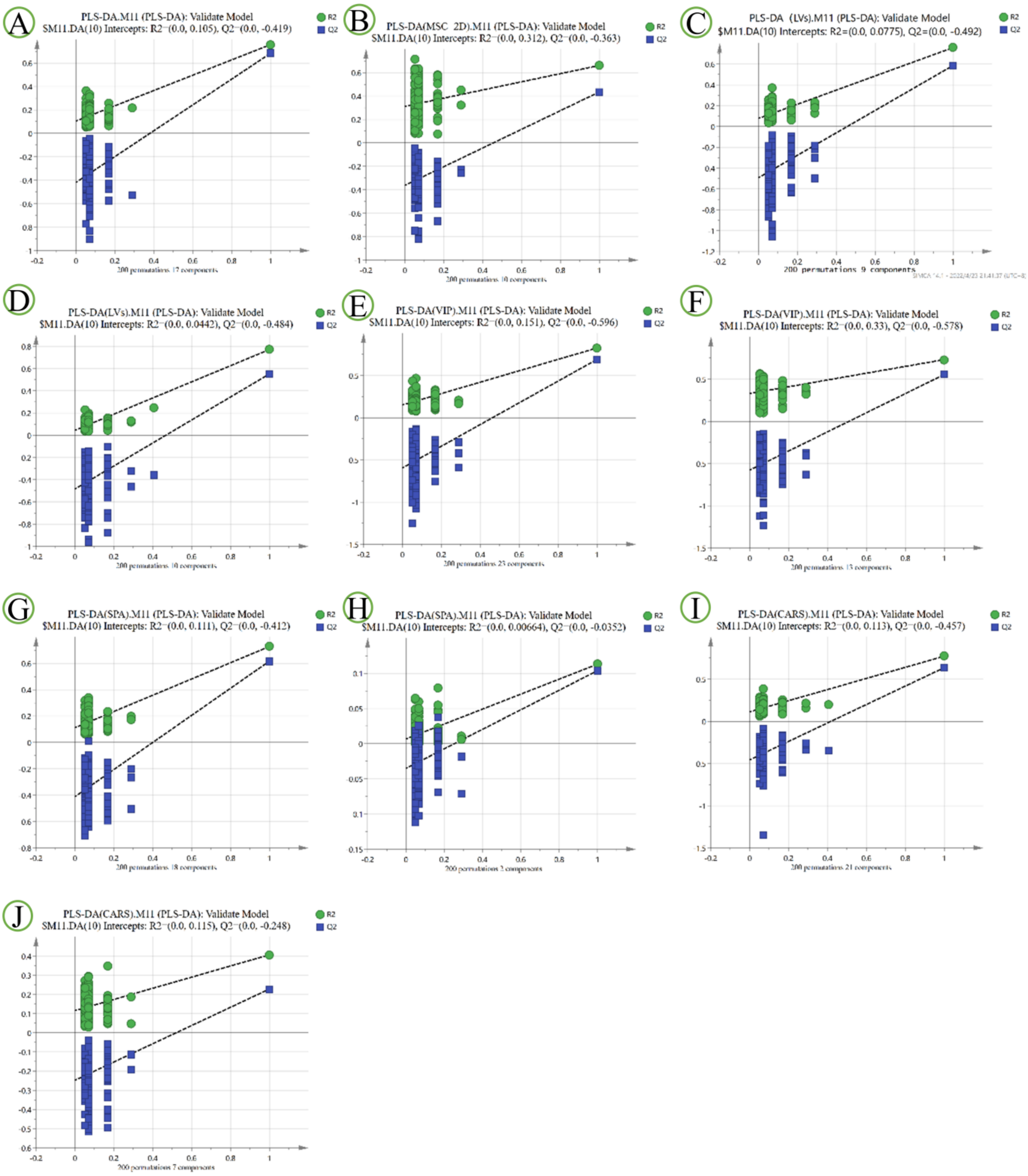

Figure S1. 200 Permutation Iteration Tests for PLS-DA Models, without preprocessing (A, C, E, G, I), MSC+SD preprocessing (B, D, F, H, J), without feature extraction (A, B), LVs (C,D), VIP (E, F), SPA (G, H), CARS (I, J).

A	Members	Acc	1	2	3	4	5	6	7	8	9	10
1	10	90%	9	0	0	1	0	0	0	0	0	0
2	13	92.31%	0	12	0	0	0	0	0	1	0	0
3	22	100%	0	0	22	0	0	0	0	0	0	0
4	21	95.24%	0	1	0	20	0	0	0	0	0	0
5	6	33.33%	0	1	0	3	2	0	0	0	0	0
6	8	100%	0	0	0	0	0	8	0	0	0	0
7	10	100%	0	0	0	0	0	0	10	0	0	0
8	15	93.33%	0	1	0	0	0	0	0	14	0	0
9	27	100%	0	0	0	0	0	0	0	0	27	0
10	9	100%	0	0	0	0	0	0	0	0	0	9
Total	141	94.33%	9	15	22	24	2	8	10	15	27	9

B	Members	Acc	1	2	3	4	5	6	7	8	9	10
1	4	75%	3	1	0	0	0	0	0	0	0	0
2	6	83.33%	0	5	0	0	0	0	0	0	1	0
3	9	88.89%	0	0	8	0	0	0	0	1	0	0
4	9	100%	0	0	0	9	0	0	0	0	0	0
5	3	0%	0	0	0	2	0	1	0	0	0	0
6	4	100%	0	0	0	0	0	4	0	0	0	0
7	4	50%	0	0	0	0	0	0	2	1	0	1
8	6	100%	0	0	0	0	0	0	0	6	0	0
9	12	100%	0	0	0	0	0	0	0	0	12	0
10	4	100%	0	0	0	0	0	0	0	0	0	4
Total	61	86.89%	3	6	8	11	0	5	2	8	13	5

C	Members	Acc	1	2	3	4	5	6	7	8	9	10
1	10	80%	8	0	0	2	0	0	0	0	0	0
2	13	100%	0	13	0	0	0	0	0	0	0	0
3	22	95.45%	0	1	21	0	0	0	0	0	0	0
4	21	90.48%	1	1	0	19	0	0	0	0	0	0
5	6	100%	0	0	0	0	6	0	0	0	0	0
6	8	100%	0	0	0	0	0	8	0	0	0	0
7	10	100%	0	0	0	0	0	0	10	0	0	0
8	15	100%	0	0	0	0	0	0	0	15	0	0
9	27	92.59%	0	1	1	0	0	0	0	0	25	0
10	9	100%	0	0	0	0	0	0	0	0	0	9
Total	141	95.04%	9	16	22	21	6	8	10	15	25	9

D	Members	Acc	1	2	3	4	5	6	7	8	9	10
1	4	75%	3	0	1	0	0	0	0	0	0	0
2	6	100%	0	6	0	0	0	0	0	0	0	0
3	9	88.89%	0	0	8	1	0	0	0	0	0	0
4	9	77.78%	0	1	0	7	1	0	0	0	0	0
5	3	66.67%	0	1	0	0	2	0	0	0	0	0
6	4	100%	0	0	0	0	0	4	0	0	0	0
7	4	75%	0	0	0	0	0	0	3	1	0	0
8	6	100%	0	0	0	0	0	0	0	6	0	0
9	12	100%	0	0	0	0	0	0	0	0	12	0
10	4	100%	0	0	0	0	0	0	0	0	0	4
Total	61	90.16%	3	8	9	8	3	4	3	7	12	4

Figure S2. Confusion matrix graph with the best performance in PLS-DA model (based on VIP feature extraction). A (training set without preprocessing), B (testing set without preprocessing), C (training set with MSC and 2D processing), and D (testing set with MSC and 2D processing).
